# (Acetonitrile-κ*N*)[2-(diphenylphosphan­yl)ethanamine-κ^2^ *N*,*P*][(1,2,3,4,5-η)-1,2,3,4,5-pentamethylcyclopentadienyl]iron(II) hexafluoridophosphate tetrahydrofuran monosolvate

**DOI:** 10.1107/S1600536811028832

**Published:** 2011-07-23

**Authors:** Kajin Lee, Frank Rominger, Michael Limbach

**Affiliations:** a CaRLa - Catalysis Research Laboratory, Im Neuenheimer Feld 584, 69120 Heidelberg, Germany; b Organisch-Chemisches Institut, Universität Heidelberg, Im Neuenheimer Feld 270, 69120 Heidelberg, Germany

## Abstract

In the title cationic Cp^*^Fe(II) complex, [Fe(C_10_H_15_)(CH_3_CN)(C_14_H_16_NP)]PF_6_·C_4_H_8_O, the metal ion is coordinated by the *η* ^5^-Cp* ring as well as the P and N atoms of the chelating 2-(diphenyl­phosphino)ethyl­amine ligand and an additional acetonitrile mol­ecule in a piano-chair conformation. The PF_6_^−^ anion is disordered over two sets of sites with occupancies of 0.779 (7) and 0.221 (7).

## Related literature

For related ruthenium complexes, see: Ito *et al.* (2003 ▶, 2005 ▶, 2007 ▶, 2009 ▶, 2011 ▶). For corresponding Fe(II) complexes, see: Davies *et al.* (1994 ▶); Lagaditis *et al.* (2010 ▶). For the structure of a similar iron complex, see: Barbier *et al.* (1979 ▶).
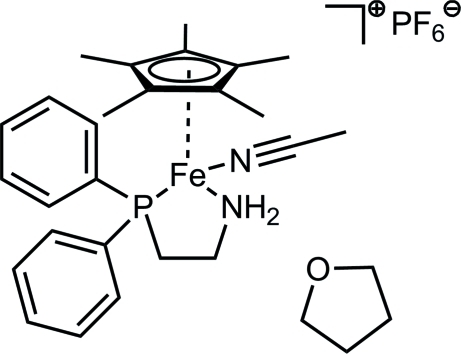


## Experimental

### 

#### Crystal data

[Fe(C_10_H_15_)(C_2_H_3_N)(C_14_H_16_NP)]PF_6_·C_4_H_8_O
*M* *_r_* = 678.45
Monoclinic, 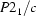

*a* = 11.9241 (2) Å
*b* = 12.9272 (1) Å
*c* = 21.4018 (4) Å
β = 103.790 (1)°
*V* = 3203.89 (8) Å^3^
*Z* = 4
Mo *K*α radiation
μ = 0.63 mm^−1^
*T* = 200 K
0.36 × 0.22 × 0.12 mm

#### Data collection

Siemens SMART CCD diffractometer
Absorption correction: multi-scan (*SADABS*; Sheldrick, 2008*a* ▶) *T*_min_ = 0.804, *T*_max_ = 0.928
32150 measured reflections
7357 independent reflections
5518 reflections with *I* > 2σ(*I*)
*R*_int_ = 0.047

#### Refinement

*R*[*F* ^2^ > 2σ(*F* ^2^)] = 0.045
*wR*(*F* ^2^) = 0.115
*S* = 1.03
7357 reflections
410 parameters
357 restraints
H-atom parameters constrained
Δρ_max_ = 0.48 e Å^−3^
Δρ_min_ = −0.52 e Å^−3^

### 

Data collection: *SMART* (Siemens, 1996 ▶); cell refinement: *SAINT* (Siemens, 1996 ▶); data reduction: *SAINT*; program(s) used to solve structure: *SHELXTL* (Sheldrick, 2008*b* ▶); program(s) used to refine structure: *SHELXTL*; molecular graphics: *SHELXTL*; software used to prepare material for publication: *SHELXTL*.

## Supplementary Material

Crystal structure: contains datablock(s) I, global. DOI: 10.1107/S1600536811028832/go2017sup1.cif

Structure factors: contains datablock(s) I. DOI: 10.1107/S1600536811028832/go2017Isup2.hkl

Additional supplementary materials:  crystallographic information; 3D view; checkCIF report

## Figures and Tables

**Fig. 1.**
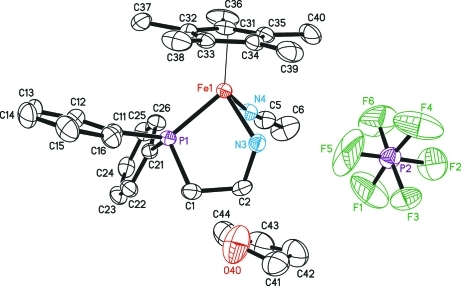
Thermal ellipsoid representation of the title compound with displacement ellipsoids plotted at 50% probability level.

## supplementary crystallographic information

### Comment

There are numerous reports on well defined Cp*Ru(II) complexes bearing a 2-(diphenylphosphino)ethylamine ligand *e.g.* by Ito *et al.* (2011), Ito *et al.* (2009), Ito *et al.* (2007), Ito *et al.* (2005), Ito *et al.* (2003). Surprisingly, only a scarce number of corresponding Fe(II) complexes has been reported, *e.g.* by Davies *et al.* (1994) and Lagaditis *et al.* (2010), although both elements are homologues in group 8. The only reported structural analysis of an iron complex with this type of P,*N*-coordination has been published by Barbier *et al.* (1979).

### Experimental

To a solution of [Cp*Fe(MeCN)_3_]PF_6_ (40 mg, 0.087 mmol) in dry MeCN was added PPh_2_(CH_2_)_2_NH_2_ (20 mg, 0.087 mmol) and the solution was stirred at 20 °C for 16 h. The solvent was removed *in vacuo*, the residue was fractionally washed with dry hexane, Et_2_O, Et_2_O/THF (1:1), THF and THF/MeCN (3:1) over a plug of celite. The last purple fraction was kept and stored at -40 °C to give purple prismatic crystals of {Cp*Fe(MeCN)[Ph_2_P(CH_2_)_2_NH_2_]}PF_6_^.^THF (14 mg, 36% yield).

### Refinement

For all hydrogen atoms the positions were calculated according to geometrical criteria. During the refinement the hydrogen atoms were allowed to shift with the preceding atoms. In the case of the methyl groups the torsion angles were allowed to refine. The isotropic displacement parameters were set as 1.2 times (1.5 for methyl) the equivalent isotropic displacement parameters of the preceding atoms. The PF_6_ anion was refined with two alternative orientations with occupancies 0.779 (7) and 0.221 (7). In order to stabilize the octahedral geometry during the refinement all equivalent bonding or nonbondig interatomic distances were restrained to be equal within a standard deviation of 0.03 Å.

### Crystal data

**Table:**

[Fe(C_10_H_15_)(C_2_H_3_N)(C_14_H_16_NP)]PF_6_·C_4_H_8_O	*F*(000) = 1416
*M**_r_* = 678.45	*D*_x_ = 1.407 Mg m^−^^3^
Monoclinic, *P*2_1_/*c*	Mo *K*α radiation, λ = 0.71073 Å
*a* = 11.9241 (2) Å	Cell parameters from 6488 reflections
*b* = 12.9272 (1) Å	µ = 0.63 mm^−^^1^
*c* = 21.4018 (4) Å	*T* = 200 K
β = 103.790 (1)°	Polyhedron, red
*V* = 3203.89 (8) Å^3^	0.36 × 0.22 × 0.12 mm
*Z* = 4	

### Data collection

**Table:**

Siemens SMART CCD diffractometer	7357 independent reflections
Radiation source: fine-focus sealed tube	5518 reflections with *I* > 2σ(*I*)
graphite	*R*_int_ = 0.047
ω scans	θ_max_ = 27.5°, θ_min_ = 1.8°
Absorption correction: multi-scan (*SADABS*; Sheldrick, 2008*a*)	*h* = −15→15
*T*_min_ = 0.804, *T*_max_ = 0.928	*k* = −16→16
32150 measured reflections	*l* = −27→27

### Refinement

**Table:**

Refinement on *F*^2^	Primary atom site location: structure-invariant direct methods
Least-squares matrix: full	Secondary atom site location: difference Fourier map
*R*[*F*^2^ > 2σ(*F*^2^)] = 0.045	Hydrogen site location: inferred from neighbouring sites
*wR*(*F*^2^) = 0.115	H-atom parameters constrained
*S* = 1.03	*w* = 1/[σ^2^(*F*_o_^2^) + (0.0463*P*)^2^ + 3.1534*P*] where *P* = (*F*_o_^2^ + 2*F*_c_^2^)/3
7357 reflections	(Δ/σ)_max_ = 0.001
410 parameters	Δρ_max_ = 0.48 e Å^−^^3^
357 restraints	Δρ_min_ = −0.52 e Å^−^^3^

### Special details

**Table:**

Geometry. All e.s.d.'s (except the e.s.d. in the dihedral angle between two l.s. planes) are estimated using the full covariance matrix. The cell e.s.d.'s are taken into account individually in the estimation of e.s.d.'s in distances, angles and torsion angles; correlations between e.s.d.'s in cell parameters are only used when they are defined by crystal symmetry. An approximate (isotropic) treatment of cell e.s.d.'s is used for estimating e.s.d.'s involving l.s. planes.
Refinement. Refinement of *F*^2^ against ALL reflections. The weighted *R*-factor *wR* and goodness of fit *S* are based on *F*^2^, conventional *R*-factors *R* are based on *F*, with *F* set to zero for negative *F*^2^. The threshold expression of *F*^2^ > σ(*F*^2^) is used only for calculating *R*-factors(gt) *etc*. and is not relevant to the choice of reflections for refinement. *R*-factors based on *F*^2^ are statistically about twice as large as those based on *F*, and *R*- factors based on ALL data will be even larger.

### Fractional atomic coordinates and isotropic or equivalent isotropic displacement parameters (Å^2^)

**Table:**

	*x*	*y*	*z*	*U*_iso_*/*U*_eq_	Occ. (<1)
Fe1	0.34034 (3)	0.71766 (3)	0.027123 (16)	0.02129 (10)	
P1	0.15826 (6)	0.76600 (5)	−0.01156 (3)	0.02342 (15)	
C1	0.1704 (2)	0.9068 (2)	−0.02448 (14)	0.0326 (6)	
H1A	0.1658	0.9210	−0.0705	0.039*	
H1B	0.1066	0.9439	−0.0119	0.039*	
C2	0.2854 (2)	0.9428 (2)	0.01627 (14)	0.0323 (6)	
H2A	0.3036	1.0125	0.0023	0.039*	
H2B	0.2819	0.9469	0.0620	0.039*	
N3	0.37661 (19)	0.86804 (17)	0.00906 (11)	0.0288 (5)	
H3A	0.4452	0.8867	0.0367	0.035*	
H3B	0.3865	0.8725	−0.0322	0.035*	
N4	0.3414 (2)	0.76209 (17)	0.11228 (11)	0.0293 (5)	
C5	0.3538 (3)	0.7830 (2)	0.16497 (14)	0.0383 (7)	
C6	0.3723 (4)	0.8075 (3)	0.23345 (16)	0.0673 (12)	
H6A	0.4201	0.7536	0.2590	0.101*	
H6B	0.2977	0.8108	0.2450	0.101*	
H6C	0.4115	0.8744	0.2422	0.101*	
C11	0.0710 (2)	0.7202 (2)	−0.08884 (12)	0.0266 (5)	
C12	−0.0186 (3)	0.6496 (2)	−0.09300 (14)	0.0348 (6)	
H12	−0.0393	0.6275	−0.0549	0.042*	
C13	−0.0779 (3)	0.6111 (3)	−0.15224 (16)	0.0449 (8)	
H13	−0.1390	0.5631	−0.1544	0.054*	
C14	−0.0491 (3)	0.6418 (3)	−0.20778 (15)	0.0484 (8)	
H14	−0.0900	0.6153	−0.2482	0.058*	
C15	0.0397 (3)	0.7114 (3)	−0.20442 (15)	0.0454 (8)	
H15	0.0603	0.7324	−0.2427	0.055*	
C16	0.0989 (3)	0.7509 (2)	−0.14581 (13)	0.0347 (6)	
H16	0.1593	0.7995	−0.1443	0.042*	
C21	0.0576 (2)	0.7543 (2)	0.04064 (12)	0.0263 (5)	
C22	−0.0415 (2)	0.8155 (2)	0.03221 (14)	0.0329 (6)	
H22	−0.0592	0.8640	−0.0021	0.040*	
C23	−0.1139 (2)	0.8057 (2)	0.07362 (15)	0.0376 (7)	
H23	−0.1802	0.8486	0.0681	0.045*	
C24	−0.0909 (3)	0.7344 (2)	0.12287 (15)	0.0381 (7)	
H24	−0.1407	0.7287	0.1514	0.046*	
C25	0.0048 (3)	0.6711 (2)	0.13068 (14)	0.0373 (7)	
H25	0.0198	0.6205	0.1638	0.045*	
C26	0.0793 (2)	0.6815 (2)	0.09000 (14)	0.0329 (6)	
H26	0.1457	0.6386	0.0960	0.040*	
C31	0.3973 (3)	0.5719 (2)	0.06174 (13)	0.0313 (6)	
C32	0.3277 (2)	0.5634 (2)	−0.00160 (14)	0.0302 (6)	
C33	0.3808 (2)	0.6228 (2)	−0.04312 (12)	0.0282 (6)	
C34	0.4835 (2)	0.6666 (2)	−0.00464 (13)	0.0283 (6)	
C35	0.4940 (2)	0.6366 (2)	0.06001 (13)	0.0310 (6)	
C36	0.3765 (4)	0.5195 (3)	0.12082 (18)	0.0594 (11)	
H36A	0.2935	0.5089	0.1157	0.089*	
H36B	0.4066	0.5631	0.1586	0.089*	
H36C	0.4161	0.4525	0.1266	0.089*	
C37	0.2230 (3)	0.4958 (2)	−0.0221 (2)	0.0565 (10)	
H37A	0.2472	0.4244	−0.0269	0.085*	
H37B	0.1757	0.5206	−0.0633	0.085*	
H37C	0.1777	0.4984	0.0105	0.085*	
C38	0.3459 (3)	0.6250 (3)	−0.11505 (14)	0.0511 (9)	
H38A	0.3633	0.6931	−0.1304	0.077*	
H38B	0.2629	0.6114	−0.1295	0.077*	
H38C	0.3887	0.5718	−0.1323	0.077*	
C39	0.5669 (3)	0.7336 (3)	−0.0293 (2)	0.0525 (9)	
H39A	0.6055	0.7812	0.0047	0.079*	
H39B	0.5248	0.7733	−0.0665	0.079*	
H39C	0.6248	0.6898	−0.0421	0.079*	
C40	0.5907 (3)	0.6656 (3)	0.11617 (17)	0.0556 (10)	
H40A	0.6533	0.6150	0.1206	0.083*	
H40B	0.5622	0.6663	0.1555	0.083*	
H40C	0.6196	0.7345	0.1090	0.083*	
P2	0.63861 (8)	0.00286 (7)	0.17490 (4)	0.0446 (2)	
F1	0.5407 (4)	0.0479 (5)	0.2052 (3)	0.134 (2)	0.779 (7)
F2	0.7248 (4)	0.0511 (3)	0.23735 (15)	0.0982 (17)	0.779 (7)
F3	0.6417 (6)	0.1097 (3)	0.13894 (19)	0.0665 (12)	0.779 (7)
F4	0.7482 (5)	−0.0333 (5)	0.1531 (3)	0.131 (2)	0.779 (7)
F5	0.5555 (7)	−0.0414 (4)	0.1165 (3)	0.190 (4)	0.779 (7)
F6	0.6392 (6)	−0.0983 (4)	0.2154 (3)	0.123 (2)	0.779 (7)
F1B	0.6358 (15)	0.0549 (9)	0.2346 (5)	0.084 (5)*	0.221 (7)
F2B	0.7666 (8)	0.0134 (10)	0.1792 (6)	0.072 (4)*	0.221 (7)
F3B	0.610 (2)	0.1033 (13)	0.1345 (11)	0.138 (14)*	0.221 (7)
F4B	0.6307 (10)	−0.0545 (9)	0.1052 (5)	0.060 (4)*	0.221 (7)
F5B	0.5069 (9)	−0.0190 (12)	0.1607 (8)	0.088 (5)*	0.221 (7)
F6B	0.6688 (14)	−0.1091 (9)	0.2030 (7)	0.064 (4)*	0.221 (7)
O40	0.1488 (3)	0.0416 (3)	0.11782 (16)	0.1004 (12)	
C41	0.2249 (4)	0.1228 (3)	0.1411 (2)	0.0727 (12)	
H41A	0.1874	0.1903	0.1281	0.087*	
H41B	0.2948	0.1177	0.1238	0.087*	
C42	0.2565 (5)	0.1126 (4)	0.2133 (2)	0.0900 (16)	
H42A	0.2638	0.1814	0.2341	0.108*	
H42B	0.3302	0.0747	0.2280	0.108*	
C43	0.1566 (4)	0.0520 (4)	0.2283 (2)	0.0813 (14)	
H43A	0.1849	0.0005	0.2627	0.098*	
H43B	0.1012	0.0988	0.2419	0.098*	
C44	0.1025 (4)	0.0001 (4)	0.1664 (2)	0.0770 (13)	
H44A	0.1177	−0.0752	0.1700	0.092*	
H44B	0.0179	0.0110	0.1559	0.092*	

### Atomic displacement parameters (Å^2^)

**Table:**

	*U*^11^	*U*^22^	*U*^33^	*U*^12^	*U*^13^	*U*^23^
Fe1	0.02332 (19)	0.02244 (18)	0.01780 (18)	0.00291 (14)	0.00426 (13)	−0.00082 (14)
P1	0.0240 (3)	0.0225 (3)	0.0237 (3)	0.0033 (3)	0.0057 (3)	0.0008 (2)
C1	0.0343 (15)	0.0261 (13)	0.0375 (15)	0.0056 (12)	0.0087 (12)	0.0044 (12)
C2	0.0411 (16)	0.0203 (13)	0.0356 (15)	0.0015 (12)	0.0096 (13)	−0.0019 (11)
N3	0.0288 (12)	0.0283 (12)	0.0282 (12)	−0.0022 (9)	0.0046 (10)	−0.0017 (9)
N4	0.0336 (13)	0.0313 (12)	0.0230 (12)	0.0054 (10)	0.0067 (9)	−0.0002 (9)
C5	0.0507 (19)	0.0382 (16)	0.0272 (15)	0.0074 (14)	0.0114 (13)	−0.0018 (13)
C6	0.114 (4)	0.065 (3)	0.0252 (17)	0.002 (2)	0.021 (2)	−0.0095 (16)
C11	0.0245 (13)	0.0273 (13)	0.0266 (13)	0.0056 (11)	0.0032 (10)	0.0005 (11)
C12	0.0360 (16)	0.0324 (15)	0.0353 (16)	−0.0007 (12)	0.0070 (13)	−0.0026 (12)
C13	0.0369 (17)	0.0409 (18)	0.052 (2)	−0.0064 (14)	0.0012 (15)	−0.0105 (15)
C14	0.0444 (19)	0.059 (2)	0.0334 (17)	0.0031 (16)	−0.0064 (14)	−0.0120 (15)
C15	0.0471 (19)	0.060 (2)	0.0260 (15)	0.0073 (16)	0.0032 (13)	0.0034 (14)
C16	0.0319 (15)	0.0409 (16)	0.0294 (15)	0.0028 (12)	0.0038 (12)	0.0033 (12)
C21	0.0262 (13)	0.0265 (13)	0.0267 (13)	0.0015 (10)	0.0070 (11)	−0.0028 (10)
C22	0.0295 (14)	0.0347 (15)	0.0333 (15)	0.0051 (12)	0.0050 (12)	0.0001 (12)
C23	0.0259 (14)	0.0448 (17)	0.0431 (17)	0.0055 (13)	0.0102 (13)	−0.0091 (14)
C24	0.0323 (15)	0.0493 (18)	0.0366 (16)	−0.0071 (13)	0.0157 (13)	−0.0110 (14)
C25	0.0408 (17)	0.0401 (16)	0.0340 (16)	−0.0002 (13)	0.0147 (13)	0.0041 (13)
C26	0.0320 (15)	0.0327 (15)	0.0357 (15)	0.0052 (12)	0.0113 (12)	0.0018 (12)
C31	0.0398 (16)	0.0273 (14)	0.0302 (14)	0.0126 (12)	0.0152 (12)	0.0071 (11)
C32	0.0261 (14)	0.0228 (13)	0.0416 (16)	0.0044 (10)	0.0080 (12)	−0.0042 (11)
C33	0.0324 (14)	0.0295 (14)	0.0218 (13)	0.0098 (11)	0.0047 (11)	−0.0034 (11)
C34	0.0266 (14)	0.0269 (13)	0.0336 (14)	0.0051 (11)	0.0114 (11)	0.0019 (11)
C35	0.0313 (14)	0.0324 (14)	0.0262 (14)	0.0100 (11)	0.0009 (11)	−0.0038 (11)
C36	0.086 (3)	0.049 (2)	0.056 (2)	0.033 (2)	0.043 (2)	0.0283 (17)
C37	0.0350 (18)	0.0300 (16)	0.103 (3)	−0.0025 (14)	0.0126 (19)	−0.0165 (18)
C38	0.070 (2)	0.057 (2)	0.0232 (15)	0.0316 (18)	0.0048 (15)	−0.0046 (14)
C39	0.0405 (19)	0.0425 (18)	0.085 (3)	0.0074 (15)	0.0354 (19)	0.0111 (18)
C40	0.0446 (19)	0.061 (2)	0.047 (2)	0.0201 (17)	−0.0171 (16)	−0.0155 (17)
P2	0.0493 (5)	0.0409 (5)	0.0442 (5)	−0.0023 (4)	0.0122 (4)	0.0065 (4)
F1	0.079 (3)	0.193 (6)	0.154 (5)	0.046 (3)	0.075 (3)	0.035 (4)
F2	0.105 (4)	0.111 (3)	0.058 (2)	0.002 (2)	−0.021 (2)	−0.0099 (19)
F3	0.098 (3)	0.0446 (18)	0.058 (2)	0.0049 (17)	0.0198 (18)	0.0229 (14)
F4	0.167 (5)	0.140 (4)	0.114 (4)	0.091 (4)	0.090 (4)	0.020 (3)
F5	0.222 (8)	0.135 (5)	0.133 (5)	−0.076 (5)	−0.114 (6)	−0.017 (4)
F6	0.166 (6)	0.076 (3)	0.139 (5)	−0.004 (3)	0.057 (4)	0.057 (3)
O40	0.113 (3)	0.129 (3)	0.066 (2)	−0.039 (2)	0.036 (2)	−0.045 (2)
C41	0.087 (3)	0.065 (3)	0.065 (3)	0.008 (2)	0.014 (2)	−0.013 (2)
C42	0.091 (4)	0.100 (4)	0.062 (3)	0.014 (3)	−0.016 (3)	−0.014 (3)
C43	0.096 (4)	0.091 (3)	0.054 (3)	0.024 (3)	0.013 (2)	0.001 (2)
C44	0.074 (3)	0.093 (4)	0.058 (3)	0.012 (3)	0.004 (2)	0.001 (2)

### Geometric parameters (Å, °)

**Table:**

Fe1—N4	1.908 (2)	C31—C36	1.506 (4)
Fe1—N3	2.048 (2)	C32—C33	1.431 (4)
Fe1—C31	2.079 (3)	C32—C37	1.501 (4)
Fe1—C32	2.082 (3)	C33—C34	1.421 (4)
Fe1—C35	2.082 (3)	C33—C38	1.496 (4)
Fe1—C33	2.084 (2)	C34—C35	1.413 (4)
Fe1—C34	2.090 (3)	C34—C39	1.507 (4)
Fe1—P1	2.2209 (7)	C35—C40	1.501 (4)
P1—C11	1.830 (3)	C36—H36A	0.9800
P1—C21	1.831 (3)	C36—H36B	0.9800
P1—C1	1.851 (3)	C36—H36C	0.9800
C1—C2	1.512 (4)	C37—H37A	0.9800
C1—H1A	0.9900	C37—H37B	0.9800
C1—H1B	0.9900	C37—H37C	0.9800
C2—N3	1.490 (3)	C38—H38A	0.9800
C2—H2A	0.9900	C38—H38B	0.9800
C2—H2B	0.9900	C38—H38C	0.9800
N3—H3A	0.9200	C39—H39A	0.9800
N3—H3B	0.9200	C39—H39B	0.9800
N4—C5	1.135 (4)	C39—H39C	0.9800
C5—C6	1.464 (4)	C40—H40A	0.9800
C6—H6A	0.9800	C40—H40B	0.9800
C6—H6B	0.9800	C40—H40C	0.9800
C6—H6C	0.9800	P2—F1	1.576 (4)
C11—C12	1.392 (4)	P2—F2	1.605 (3)
C11—C16	1.396 (4)	P2—F3	1.586 (3)
C12—C13	1.388 (4)	P2—F4	1.561 (4)
C12—H12	0.9500	P2—F5	1.511 (4)
C13—C14	1.372 (5)	P2—F6	1.569 (4)
C13—H13	0.9500	P2—F1B	1.451 (9)
C14—C15	1.378 (5)	P2—F2B	1.513 (10)
C14—H14	0.9500	P2—F3B	1.553 (12)
C15—C16	1.382 (4)	P2—F4B	1.647 (9)
C15—H15	0.9500	P2—F5B	1.553 (10)
C16—H16	0.9500	P2—F6B	1.576 (11)
C21—C26	1.392 (4)	O40—C44	1.394 (5)
C21—C22	1.397 (4)	O40—C41	1.400 (5)
C22—C23	1.382 (4)	C41—C42	1.506 (6)
C22—H22	0.9500	C41—H41A	0.9900
C23—C24	1.378 (4)	C41—H41B	0.9900
C23—H23	0.9500	C42—C43	1.523 (7)
C24—C25	1.382 (4)	C42—H42A	0.9900
C24—H24	0.9500	C42—H42B	0.9900
C25—C26	1.390 (4)	C43—C44	1.489 (6)
C25—H25	0.9500	C43—H43A	0.9900
C26—H26	0.9500	C43—H43B	0.9900
C31—C32	1.415 (4)	C44—H44A	0.9900
C31—C35	1.433 (4)	C44—H44B	0.9900
			
N4—Fe1—N3	86.62 (9)	C33—C32—Fe1	69.97 (15)
N4—Fe1—C31	90.30 (10)	C37—C32—Fe1	129.8 (2)
N3—Fe1—C31	149.53 (11)	C34—C33—C32	107.6 (2)
N4—Fe1—C32	123.64 (11)	C34—C33—C38	125.3 (3)
N3—Fe1—C32	149.00 (10)	C32—C33—C38	126.4 (3)
C31—Fe1—C32	39.76 (11)	C34—C33—Fe1	70.32 (14)
N4—Fe1—C35	91.36 (10)	C32—C33—Fe1	69.84 (14)
N3—Fe1—C35	109.44 (10)	C38—C33—Fe1	132.53 (19)
C31—Fe1—C35	40.28 (11)	C35—C34—C33	108.8 (2)
C32—Fe1—C35	67.29 (11)	C35—C34—C39	126.0 (3)
N4—Fe1—C33	156.39 (10)	C33—C34—C39	125.2 (3)
N3—Fe1—C33	109.08 (10)	C35—C34—Fe1	69.93 (15)
C31—Fe1—C33	67.13 (10)	C33—C34—Fe1	69.87 (14)
C32—Fe1—C33	40.18 (11)	C39—C34—Fe1	126.2 (2)
C35—Fe1—C33	67.14 (10)	C34—C35—C31	107.5 (2)
N4—Fe1—C34	125.88 (10)	C34—C35—C40	126.0 (3)
N3—Fe1—C34	90.75 (10)	C31—C35—C40	126.5 (3)
C31—Fe1—C34	66.83 (10)	C34—C35—Fe1	70.47 (15)
C32—Fe1—C34	66.94 (11)	C31—C35—Fe1	69.73 (15)
C35—Fe1—C34	39.60 (10)	C40—C35—Fe1	126.2 (2)
C33—Fe1—C34	39.81 (11)	C31—C36—H36A	109.5
N4—Fe1—P1	93.13 (7)	C31—C36—H36B	109.5
N3—Fe1—P1	83.93 (7)	H36A—C36—H36B	109.5
C31—Fe1—P1	126.53 (9)	C31—C36—H36C	109.5
C32—Fe1—P1	99.44 (8)	H36A—C36—H36C	109.5
C35—Fe1—P1	166.14 (8)	H36B—C36—H36C	109.5
C33—Fe1—P1	105.69 (8)	C32—C37—H37A	109.5
C34—Fe1—P1	140.31 (8)	C32—C37—H37B	109.5
C11—P1—C21	102.13 (12)	H37A—C37—H37B	109.5
C11—P1—C1	103.34 (13)	C32—C37—H37C	109.5
C21—P1—C1	104.98 (12)	H37A—C37—H37C	109.5
C11—P1—Fe1	122.73 (9)	H37B—C37—H37C	109.5
C21—P1—Fe1	118.32 (9)	C33—C38—H38A	109.5
C1—P1—Fe1	103.18 (9)	C33—C38—H38B	109.5
C2—C1—P1	107.98 (18)	H38A—C38—H38B	109.5
C2—C1—H1A	110.1	C33—C38—H38C	109.5
P1—C1—H1A	110.1	H38A—C38—H38C	109.5
C2—C1—H1B	110.1	H38B—C38—H38C	109.5
P1—C1—H1B	110.1	C34—C39—H39A	109.5
H1A—C1—H1B	108.4	C34—C39—H39B	109.5
N3—C2—C1	108.9 (2)	H39A—C39—H39B	109.5
N3—C2—H2A	109.9	C34—C39—H39C	109.5
C1—C2—H2A	109.9	H39A—C39—H39C	109.5
N3—C2—H2B	109.9	H39B—C39—H39C	109.5
C1—C2—H2B	109.9	C35—C40—H40A	109.5
H2A—C2—H2B	108.3	C35—C40—H40B	109.5
C2—N3—Fe1	113.80 (17)	H40A—C40—H40B	109.5
C2—N3—H3A	108.8	C35—C40—H40C	109.5
Fe1—N3—H3A	108.8	H40A—C40—H40C	109.5
C2—N3—H3B	108.8	H40B—C40—H40C	109.5
Fe1—N3—H3B	108.8	F5—P2—F1	94.4 (4)
H3A—N3—H3B	107.7	F6—P2—F1	89.2 (3)
C5—N4—Fe1	172.2 (2)	F4—P2—F1	171.6 (3)
N4—C5—C6	178.3 (4)	F4—P2—F2	87.1 (3)
C5—C6—H6A	109.5	F6—P2—F2	86.9 (3)
C5—C6—H6B	109.5	F1—P2—F2	84.5 (3)
H6A—C6—H6B	109.5	F3—P2—F2	88.9 (3)
C5—C6—H6C	109.5	F5—P2—F2	178.9 (4)
H6A—C6—H6C	109.5	F5—P2—F3	90.8 (3)
H6B—C6—H6C	109.5	F4—P2—F3	89.9 (3)
C12—C11—C16	118.0 (3)	F1—P2—F3	89.0 (3)
C12—C11—P1	122.2 (2)	F6—P2—F3	175.6 (3)
C16—C11—P1	119.6 (2)	F5—P2—F4	94.0 (4)
C13—C12—C11	120.6 (3)	F5—P2—F6	93.3 (3)
C13—C12—H12	119.7	F4—P2—F6	91.3 (3)
C11—C12—H12	119.7	F1B—P2—F2B	98.0 (7)
C14—C13—C12	120.6 (3)	F1B—P2—F3B	93.1 (10)
C14—C13—H13	119.7	F2B—P2—F3B	92.6 (10)
C12—C13—H13	119.7	F5B—P2—F4B	84.4 (7)
C13—C14—C15	119.4 (3)	F6B—P2—F4B	83.7 (7)
C13—C14—H14	120.3	F2B—P2—F4B	86.3 (6)
C15—C14—H14	120.3	F3B—P2—F4B	85.1 (10)
C14—C15—C16	120.6 (3)	F1B—P2—F4B	175.4 (8)
C14—C15—H15	119.7	F1B—P2—F5B	91.3 (8)
C16—C15—H15	119.7	F3B—P2—F5B	87.9 (10)
C15—C16—C11	120.7 (3)	F2B—P2—F5B	170.6 (8)
C15—C16—H16	119.6	F1B—P2—F6B	98.2 (7)
C11—C16—H16	119.6	F2B—P2—F6B	85.6 (7)
C26—C21—C22	118.6 (2)	F5B—P2—F6B	92.1 (7)
C26—C21—P1	119.2 (2)	F3B—P2—F6B	168.7 (11)
C22—C21—P1	122.1 (2)	C44—O40—C41	111.2 (3)
C23—C22—C21	120.2 (3)	O40—C41—C42	106.3 (4)
C23—C22—H22	119.9	O40—C41—H41A	110.5
C21—C22—H22	119.9	C42—C41—H41A	110.5
C24—C23—C22	120.7 (3)	O40—C41—H41B	110.5
C24—C23—H23	119.6	C42—C41—H41B	110.5
C22—C23—H23	119.6	H41A—C41—H41B	108.7
C23—C24—C25	119.8 (3)	C41—C42—C43	104.2 (4)
C23—C24—H24	120.1	C41—C42—H42A	110.9
C25—C24—H24	120.1	C43—C42—H42A	110.9
C24—C25—C26	120.0 (3)	C41—C42—H42B	110.9
C24—C25—H25	120.0	C43—C42—H42B	110.9
C26—C25—H25	120.0	H42A—C42—H42B	108.9
C25—C26—C21	120.6 (3)	C44—C43—C42	103.7 (4)
C25—C26—H26	119.7	C44—C43—H43A	111.0
C21—C26—H26	119.7	C42—C43—H43A	111.0
C32—C31—C35	108.2 (2)	C44—C43—H43B	111.0
C32—C31—C36	126.4 (3)	C42—C43—H43B	111.0
C35—C31—C36	125.4 (3)	H43A—C43—H43B	109.0
C32—C31—Fe1	70.23 (15)	O40—C44—C43	109.0 (4)
C35—C31—Fe1	69.99 (15)	O40—C44—H44A	109.9
C36—C31—Fe1	127.0 (2)	C43—C44—H44A	109.9
C31—C32—C33	107.9 (2)	O40—C44—H44B	109.9
C31—C32—C37	125.6 (3)	C43—C44—H44B	109.9
C33—C32—C37	126.2 (3)	H44A—C44—H44B	108.3
C31—C32—Fe1	70.01 (15)		
			
N4—Fe1—P1—C11	−163.17 (13)	N4—Fe1—C32—C37	82.4 (3)
N3—Fe1—P1—C11	110.57 (12)	N3—Fe1—C32—C37	−111.7 (3)
C31—Fe1—P1—C11	−70.48 (14)	C31—Fe1—C32—C37	120.2 (4)
C32—Fe1—P1—C11	−38.32 (13)	C35—Fe1—C32—C37	158.0 (3)
C35—Fe1—P1—C11	−54.5 (4)	C33—Fe1—C32—C37	−121.0 (4)
C33—Fe1—P1—C11	2.41 (13)	C34—Fe1—C32—C37	−158.8 (3)
C34—Fe1—P1—C11	26.75 (16)	P1—Fe1—C32—C37	−17.8 (3)
N4—Fe1—P1—C21	−34.11 (12)	C31—C32—C33—C34	0.5 (3)
N3—Fe1—P1—C21	−120.37 (12)	C37—C32—C33—C34	−174.2 (3)
C31—Fe1—P1—C21	58.58 (14)	Fe1—C32—C33—C34	60.50 (17)
C32—Fe1—P1—C21	90.74 (13)	C31—C32—C33—C38	171.4 (3)
C35—Fe1—P1—C21	74.6 (3)	C37—C32—C33—C38	−3.3 (4)
C33—Fe1—P1—C21	131.47 (12)	Fe1—C32—C33—C38	−128.6 (3)
C34—Fe1—P1—C21	155.81 (15)	C31—C32—C33—Fe1	−59.97 (18)
N4—Fe1—P1—C1	81.21 (12)	C37—C32—C33—Fe1	125.3 (3)
N3—Fe1—P1—C1	−5.05 (12)	N4—Fe1—C33—C34	−62.6 (3)
C31—Fe1—P1—C1	173.91 (13)	N3—Fe1—C33—C34	66.82 (16)
C32—Fe1—P1—C1	−153.94 (13)	C31—Fe1—C33—C34	−80.73 (17)
C35—Fe1—P1—C1	−170.1 (3)	C32—Fe1—C33—C34	−118.2 (2)
C33—Fe1—P1—C1	−113.20 (13)	C35—Fe1—C33—C34	−36.85 (16)
C34—Fe1—P1—C1	−88.87 (15)	P1—Fe1—C33—C34	155.73 (13)
C11—P1—C1—C2	−148.80 (19)	N4—Fe1—C33—C32	55.6 (3)
C21—P1—C1—C2	104.5 (2)	N3—Fe1—C33—C32	−174.97 (15)
Fe1—P1—C1—C2	−20.0 (2)	C31—Fe1—C33—C32	37.47 (16)
P1—C1—C2—N3	44.9 (3)	C35—Fe1—C33—C32	81.36 (17)
C1—C2—N3—Fe1	−53.3 (3)	C34—Fe1—C33—C32	118.2 (2)
N4—Fe1—N3—C2	−61.81 (18)	P1—Fe1—C33—C32	−86.07 (15)
C31—Fe1—N3—C2	−146.6 (2)	N4—Fe1—C33—C38	177.0 (3)
C32—Fe1—N3—C2	129.9 (2)	N3—Fe1—C33—C38	−53.5 (3)
C33—Fe1—N3—C2	136.24 (18)	C31—Fe1—C33—C38	158.9 (3)
C34—Fe1—N3—C2	172.29 (19)	C32—Fe1—C33—C38	121.4 (4)
P1—Fe1—N3—C2	31.71 (17)	C35—Fe1—C33—C38	−157.2 (3)
C35—Fe1—N4—C5	3.7 (19)	C34—Fe1—C33—C38	−120.4 (4)
P1—Fe1—N4—C5	170.6 (19)	P1—Fe1—C33—C38	35.4 (3)
C21—P1—C11—C12	−27.8 (3)	C32—C33—C34—C35	−0.9 (3)
C1—P1—C11—C12	−136.6 (2)	C38—C33—C34—C35	−171.9 (3)
Fe1—P1—C11—C12	107.9 (2)	Fe1—C33—C34—C35	59.30 (18)
C21—P1—C11—C16	156.7 (2)	C32—C33—C34—C39	179.0 (3)
C1—P1—C11—C16	47.8 (2)	C38—C33—C34—C39	8.0 (4)
Fe1—P1—C11—C16	−67.7 (2)	Fe1—C33—C34—C39	−120.8 (3)
C16—C11—C12—C13	0.0 (4)	C32—C33—C34—Fe1	−60.20 (17)
P1—C11—C12—C13	−175.6 (2)	C38—C33—C34—Fe1	128.8 (3)
C11—C12—C13—C14	0.2 (5)	N4—Fe1—C34—C35	34.1 (2)
C12—C13—C14—C15	0.0 (5)	N3—Fe1—C34—C35	120.42 (17)
C13—C14—C15—C16	−0.5 (5)	C31—Fe1—C34—C35	−38.33 (17)
C14—C15—C16—C11	0.8 (5)	C32—Fe1—C34—C35	−81.74 (18)
C12—C11—C16—C15	−0.5 (4)	C33—Fe1—C34—C35	−119.9 (2)
P1—C11—C16—C15	175.2 (2)	P1—Fe1—C34—C35	−158.20 (13)
C11—P1—C21—C26	112.6 (2)	N4—Fe1—C34—C33	153.96 (15)
C1—P1—C21—C26	−139.8 (2)	N3—Fe1—C34—C33	−119.67 (16)
Fe1—P1—C21—C26	−25.5 (3)	C31—Fe1—C34—C33	81.57 (17)
C11—P1—C21—C22	−66.8 (2)	C32—Fe1—C34—C33	38.17 (16)
C1—P1—C21—C22	40.8 (3)	C35—Fe1—C34—C33	119.9 (2)
Fe1—P1—C21—C22	155.1 (2)	P1—Fe1—C34—C33	−38.3 (2)
C26—C21—C22—C23	2.1 (4)	N4—Fe1—C34—C39	−86.5 (3)
P1—C21—C22—C23	−178.5 (2)	N3—Fe1—C34—C39	−0.2 (3)
C21—C22—C23—C24	−1.3 (4)	C31—Fe1—C34—C39	−158.9 (3)
C22—C23—C24—C25	−0.6 (5)	C32—Fe1—C34—C39	157.7 (3)
C23—C24—C25—C26	1.8 (5)	C35—Fe1—C34—C39	−120.6 (3)
C24—C25—C26—C21	−1.0 (5)	C33—Fe1—C34—C39	119.5 (3)
C22—C21—C26—C25	−1.0 (4)	P1—Fe1—C34—C39	81.2 (3)
P1—C21—C26—C25	179.6 (2)	C33—C34—C35—C31	0.9 (3)
N4—Fe1—C31—C32	149.28 (17)	C39—C34—C35—C31	−179.0 (3)
N3—Fe1—C31—C32	−126.9 (2)	Fe1—C34—C35—C31	60.17 (18)
C35—Fe1—C31—C32	−119.0 (2)	C33—C34—C35—C40	179.6 (3)
C33—Fe1—C31—C32	−37.86 (16)	C39—C34—C35—C40	−0.3 (5)
C34—Fe1—C31—C32	−81.28 (17)	Fe1—C34—C35—C40	−121.2 (3)
P1—Fe1—C31—C32	55.17 (18)	C33—C34—C35—Fe1	−59.27 (18)
N4—Fe1—C31—C35	−91.74 (16)	C39—C34—C35—Fe1	120.8 (3)
N3—Fe1—C31—C35	−7.9 (3)	C32—C31—C35—C34	−0.6 (3)
C32—Fe1—C31—C35	119.0 (2)	C36—C31—C35—C34	177.6 (3)
C33—Fe1—C31—C35	81.11 (16)	Fe1—C31—C35—C34	−60.64 (18)
C34—Fe1—C31—C35	37.70 (15)	C32—C31—C35—C40	−179.2 (3)
P1—Fe1—C31—C35	174.14 (12)	C36—C31—C35—C40	−1.1 (5)
N4—Fe1—C31—C36	28.0 (3)	Fe1—C31—C35—C40	120.7 (3)
N3—Fe1—C31—C36	111.9 (3)	C32—C31—C35—Fe1	60.08 (18)
C32—Fe1—C31—C36	−121.2 (4)	C36—C31—C35—Fe1	−121.8 (3)
C35—Fe1—C31—C36	119.8 (4)	N4—Fe1—C35—C34	−153.01 (17)
C33—Fe1—C31—C36	−159.1 (3)	N3—Fe1—C35—C34	−66.11 (17)
C34—Fe1—C31—C36	157.5 (3)	C31—Fe1—C35—C34	118.1 (2)
P1—Fe1—C31—C36	−66.1 (3)	C32—Fe1—C35—C34	80.78 (17)
C35—C31—C32—C33	0.0 (3)	C33—Fe1—C35—C34	37.03 (16)
C36—C31—C32—C33	−178.1 (3)	P1—Fe1—C35—C34	98.1 (4)
Fe1—C31—C32—C33	59.94 (18)	N4—Fe1—C35—C31	88.87 (16)
C35—C31—C32—C37	174.8 (3)	N3—Fe1—C35—C31	175.76 (15)
C36—C31—C32—C37	−3.3 (4)	C32—Fe1—C35—C31	−37.34 (16)
Fe1—C31—C32—C37	−125.3 (3)	C33—Fe1—C35—C31	−81.10 (17)
C35—C31—C32—Fe1	−59.93 (18)	C34—Fe1—C35—C31	−118.1 (2)
C36—C31—C32—Fe1	121.9 (3)	P1—Fe1—C35—C31	−20.0 (4)
N4—Fe1—C32—C31	−37.8 (2)	N4—Fe1—C35—C40	−32.1 (3)
N3—Fe1—C32—C31	128.0 (2)	N3—Fe1—C35—C40	54.8 (3)
C35—Fe1—C32—C31	37.82 (16)	C31—Fe1—C35—C40	−121.0 (4)
C33—Fe1—C32—C31	118.8 (2)	C32—Fe1—C35—C40	−158.3 (3)
C34—Fe1—C32—C31	80.96 (17)	C33—Fe1—C35—C40	157.9 (3)
P1—Fe1—C32—C31	−138.04 (15)	C34—Fe1—C35—C40	120.9 (4)
N4—Fe1—C32—C33	−156.62 (15)	P1—Fe1—C35—C40	−141.0 (3)
N3—Fe1—C32—C33	9.3 (3)	C44—O40—C41—C42	17.5 (5)
C31—Fe1—C32—C33	−118.8 (2)	O40—C41—C42—C43	−23.4 (5)
C35—Fe1—C32—C33	−80.96 (16)	C41—C42—C43—C44	20.5 (5)
C34—Fe1—C32—C33	−37.82 (15)	C41—O40—C44—C43	−3.9 (6)
P1—Fe1—C32—C33	103.18 (14)	C42—C43—C44—O40	−11.0 (5)
